# The combination of *Cassia obtusifolia* L. and *Foeniculum vulgare* M. exhibits a laxative effect on loperamide-induced constipation of rats

**DOI:** 10.1371/journal.pone.0195624

**Published:** 2018-04-05

**Authors:** Seung Hee Jang, Dong Kwon Yang

**Affiliations:** 1 Research & Development in TEAZEN, Inc., Anyang-si, Gyeonggi-do, Republic of Korea; 2 Department of Veterinary Pharmacology and Toxicology, College of Veterinary Medicine, Chonbuk National University, Gobong-ro, Iksan-si, Jeollabuk-do, Republic of Korea; Sungkyunkwan University, REPUBLIC OF KOREA

## Abstract

Chronic constipation is a functional gastrointestinal disease that is detrimental to the quality of patient life. *Cassia obtusifolia* L. (CO) and *Foeniculum vulgare* M. (FV) are commonly used as medicinal foods in many countries. We aimed to examine the laxative effect and their underlying mechanism of CO and FV mixture on loperamide (lop)-induced constipated rats. To determine the laxative effects of these compounds, Sprague-Dawley rats were divided into six groups: the control, lop-induced constipated (2mg/kg), and three doses (100, 300, and 500mg/kg) of CO and FV mixture-, and Bisacodyl (bis, 3.3mg/kg)-treated groups. The mixture of CO and FV and bis were orally administered once a day for 4 weeks. For induction of constipation, the lop were treated with a dose of 2 mg/kg twice a day on the 3^rd^ week after treatments of CO and FV extracts and bis. The results were revealed that the CO and FV mixture has the laxative effects more than those in CO and FV-alone treatments on constipated rats by determining the stool parameters, including stool number and weight. Indeed, stool parameters, such as, stool number, weight, and water contents and colonic peristalsis from the intestinal transit length and ratio were dramatically improved by CO and FV mixture treatment. Histological study also revealed that CO and FV mixture enhanced the thicknesses of mucosa and muscular layers of the colon in constipated rats. For their underlying mechanism, the mRNAs and proteins expression of muscarinic acetylcholine receptors (mAchR) M2 and M3 and their downstream signaling were preserved by CO and FV mixture treatment in constipated rats. Therefore, this study suggests that treatment with CO and FV mixture has beneficial effects against constipation. We further suggest that CO and FV mixture may be utilized as an alternative therapeutic strategy for constipation.

## Introduction

Constipation is one of the most frequent functional gastrointestinal disorder [1, 2]. The prevalence of chronic constipation is 3–15% of the global population, especially in the elderly over 65 years of age and women [3]. This disease is often caused by metabolic imbalance, inadequate fluid intake, medication, and fiber deficiency [4]. The main symptoms of chronic constipation include infrequent bowel movements, reducing the quantity of feces, dry stools, and difficulty with defecation [5]. Chronic constipation may have deleterious effects on the quality of patients’ life and increases their economic cost. Therefore, medical treatments or therapeutic interventions are needed to treat and prevent the chronic constipation.

Currently, various therapeutic approaches are commonly used to treat the constipation. Commonly used methods for treatment of constipation include supplement of fibers from the diet, plenty of fluids, enemas, and stimulants [6]. However, because of their low-efficacy, the medical needs should be required for mitigating the symptoms of constipation [7]. At present, there are three typical prescription drugs, such as linaclotide, lubiprostone, and naloxegol. Linaclotide is a guanylate cyclase C receptor agonist that inhibits visceral pain and stimulates intestinal motility [8]. Lubiprostone is a prostaglandin E analog that increases osmotic pressure in the intestinal lumen by activating the lubiprostone-induced type-2 chloride channel (CIC-2) and cystic fibrosis transmembrane conductance regulator (CFTR) [9]. Naloxegol is a μ-opioid receptor antagonist that is used for opioid-induced constipation [10]. Due to their less effective and side effects on the patients, there is a need for more effective and safe drugs to treat constipation.

Regarding with the underlying mechanism of constipation, mAchR M2, M3 and their downstream signaling pathway, including PKC and PI3K proteins have been reported to have crucial roles for the contraction of smooth muscle cells [11]. Indeed, previous studies have shown to alter the levels of these mAchR M2, M3, and their downstream signaling mRNA and proteins in constipation [12, 13]. Therefore, these proteins are considered to be important factors for evaluating the laxative effects of drugs against constipation.

Recently, herbal plants have been identified as new targets for the treatment of constipation. Of these, *Malva sylvestris* L., known as a common mallow, is an herbal plant, which has been reported to have potent laxative and antioxidant properties in constipation treatment [14]. In addition, *Aloe ferox* Mill., belongs to the Ashodelaceae family, which has laxative properties, such as improvement of intestinal motility and increase of fecal volume in the constipated rats [15].

*Cassia obtusifolia* L. (CO) belongs to the Leguminosae family (subfamily Caesalpinoideae) and is widely used as a traditional herbal medicine [16]. Previous studies have demonstrated that it has numerous biological activities including hepatoprotective [17], neuroprotective [18], and anti-microbial [19] activities. Furthermore, it has also been reported to have improving actions for the ulcerative colitis by regulating the chronic intestinal inflammation [20]. In addition, the major active components of *C*. *obtusifolia*, such as anthraquinones, naphthopyrones, and lactones, have been identified [21].

*Foeniculum vulgare* M. (FV) commonly called fennel is a medicinal plant belongs to the Apiaceae family. This Fennel plants have been traditionally used as a source of folk medicine for the treatment of digestive, diuretic, respiratory, and gastrointestinal disorders [22]. Previous studies have demonstrated that fennel essential oil exhibits the anti-fungal [23], anti-bacterial [24], and anti-inflammatory [25] activities from *in vitro* and *in vivo* approaches. FV contains many flavonoids (such as quercetin, isoquercetin, and kampferol) and phenolic compounds, including galliac acid, p-coumaric acid, and chlorogenic acid [26]. The laxative activities of these plants against constipation are still unknown, although various studies have demonstrated their beneficial effects on many diseases.

Therefore, the present study was designed to investigate the laxative potential of combination of CO and FV in loperamide (lop)-induced constipated rats. We concluded that combination of these extracts strongly alleviates the symptoms of constipation.

## Materials and methods

### Ethics statement

All animal experiments in this study were approved by the Animal Care Committee of Wonkang University (Approval number: WKU16-83) and was performed according to the guidelines from Wonkang University IACUC and the NIH principles for the Care and Use of Laboratory Animals.

### Preparation of CO and FV

The seed extracts of CO (NAT-041) and FV (NAT-096) were purchased from the Naturalin Bio-Resources Co., Ltd (Changsha, China). Briefly, the seeds of CO and FV were dried in an incubator at 60°C and were powdered in an electric blender. The dried CO and FV powders were then twice extracted in 70% ethanol (powder sample/70% ethanol, 1:8) at 70°C for 3 h. The extracts were filtered and evaporated in a rotary evaporator. The seed extracts were then validated by analysis of Aurantio-obusin and miquelianin contents as marker compounds for CO and FV using HPLC. For the mixture of CO and FV extracts, each extract was mixed together in a 1:1 ratio.

### Animal study design and induction of constipation

The 6 weeks-aged (weight 150–200 g) male Sprague-Dawley rats (Samtako Biokorea, Daejeon, Korea) were used for all experiments. Animals were housed in cages maintained at 23 ± 2°C with 50 ± 10% humidity on a 12 h light:dark schedule. The rats were divided into 6 groups: control group, lop-induced constipated group, lop-treated with administration of 100, 300, and 500 mg/kg CO and FV mixture group, and bisacodyl (bis)-treated group (n = 10 in each group). For CO and FV alone treatment, each Co and FV extract was daily administered by oral gavage with a dose of 300 mg/kg for 4 weeks. CO and FV mixture was also daily administered with a dose of 100, 300, and 500mg/kg for 4 weeks, respectively. Finally, for the bis-treated group as a positive control group, bis was orally administered once a day with a dose of 3.3mg/kg/day. For induction of constipation, the rats were orally administered 2mg/kg of lop in 0.9% sodium chloride twice a day for 1week on the 3^rd^-week after starting the administration of CO, FV extracts and bis. For euthanasia, the rats were anesthetized by CO2 inhalation to minimize suffering.

### Analysis of body weight, food intake, and water consumption

The body weight, food intake, and water consumption of rats in each group was daily measured during the experimental period. All measurements were performed in triplicate to ensure accuracy.

### Measurement of stool parameters

The excreted fecal pellets were daily collected throughout the experiment. Fecal samples were then weighed and counted three times for the analysis of stool number and weight. For analysis of stool water content, feces samples were dried at 40°C for 24 h and weighed. Stool water content was then determined by subtracting dried feces from wet feces.

### Measurement of barium sulfate intestine transit length and ratio

The determination of gastrointestinal propulsion of the barium sulfate was performed as described previously with minor modifications [27]. Briefly, the rats were fasted for 12 h before administration of barium sulfate, and they were then orally received the 2 ml of barium sulfate (1.4g/ml; Dongin-dang pharmaceutical Co., Ltd., Siheung, Korea). At 1 h after treatment of barium sulfate, the rats were sacrificed and the small intestines in each group were rapidly dissected. Thereafter, the distance moved by the barium sulfate was measured for the analysis of intestine transit length. For the analysis of intestine transit ratio, it was calculated by the following formula: Intestine transit ratio (%) = distance moved by the barium sulfate (cm)/total intestine length (cm) X 100.

### Histological analysis of the transverse colon

The rats were euthanized and the transverse colons in each group were rapidly removed and fixed with 10% neutral-buffered formalin. After fixation, the tissues were embedded in paraffin, and embedded tissues were cut into 5-μm thick sections. The sections were then stained with hematoxylin-eosin (H&E) and examined using light microscopy (Carl Zeiss, Jena, Germany). The thicknesses of mucosa and muscular layers in the transverse colon were measured by AnalySIS 2.3 software (Carl Zeiss).

### Quantitative real-time PCR (qRT-PCR)

Total RNA was isolated from the frozen tissue of transverse colon by using Ribospin^TM^ II kit (GeneAll biotechnology Co., LTD, Seoul, Korea). 1μg of total RNA in each group was applied to reverse transcription PCR by using ImProm II Reverse Transcriptase (Promega, Medison, USA) with oligo-dT priming. qRT-PCR was conducted using a TaKaRa Thermal Cycler Dice Real Time System Single TP 815 (Takara, Shiga, Japan) with SYBR Green (Takara) used to examine the mRNA expression of mAchR M2 and M3 in the colon. The primer sequences were as follows: mAchR M2 forward: 5'- TCC CGG GCA AGC AAG AGT AG -3', reverse: 5'- CCA TCA CCA CCA GGC ATA TTG TTA -3'; mAchR M3 forward: 5'- GCA AAG CTG ACA CCA CTT GTC -3', reverse: 5'- GTG TGA AAC TTG AAC AGC ACG AAA C-3'; 18S forward: 5'- TTC TGG CCA ACG GTC TAG ACA AC-3', 18S reverse: 5'- CCA GTG GTC TTG GTG TGC TGA -3'.

### Western blot analysis

Protein samples were prepared from the transverse colon using RIPA lysis buffer supplemented with protease inhibitor cocktail (Roche Dignostics, Mannheim, Germany) and phosphatase inhibitor cocktail (Roche Dignostics). Protein samples were separated on SDS-PAGE gels and transferred to PVDF membranes (Millipore, Billerica, USA). After 2 h blocking with 5% BSA (Sigma-Aldrich, St Louis, USA), the membranes were incubated overnight at 4°C with the following antibodies: mAchR M2 (Alomones Labs, Jerusalem, Israel), mAchR M3 (Alomones Labs), phosphorylated PKC (Cell Signaling Technology, Danvers, USA), PKC (Cell Signaling Technology), phosphorylated PI3K (Cell Signaling Technology), PI3K (Cell Signaling Technology), and β-actin (Santa Cruz Biotechnology, Santa Cruz, USA). Next, the membranes were incubated with the horseradish peroxidase (HRP)-conjugated secondary antibodies (Cell Signaling Technology) at room temperature for 1 h and detected by using an Immobilon Western Chemiluminescence kit (Millipore Corp., Billerica, USA) and UVITEC Mini HD9 (Cleaver Scientific Ltd., Warwickshire, UK).

### Statistical analysis

All data are reported as the mean ± SEM. Statistical significance was analyzed using one-way ANOVA with Bonferroni post-hoc test (Prism 5.0.3, GraphPad Software Inc., San Diego, USA). *P* <0.05 was considered statistically significant.

## Results

### Effects of CO, FV, and combined treatment on lop-induced constipated rats

**Fig 1:**
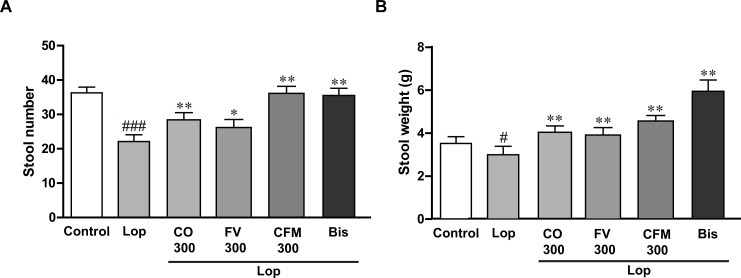
The laxative effects of CO and FV treatment on lop-induced constipated rats. Stool number (A), stool weight (B) daily measured in each group. N = 10 in each group. Data are expressed as mean ± SEM. Significance was measured by performing a one-way ANOVA followed by Bonferroni’s post-hoc test. ^#^ P < 0.05 and ^##^ P < 0.01 vs. control. * P < 0.05, ** P < 0.01, and *** P < 0.001 vs. Lop-treated group. Lop, loperamide-induced constipated group; CO300, CO 300mg/kg/day-treated group; FV300, FV 300mg/kg/day-treated group; CFM300, CO and FV mixture 300 mg/kg/day-treated group, respectively; Bis, bisacodyl-treated group.

To test the laxative effects of CO, FV, and their combined treatments on lop-induced constipated rats, the fecal parameters, including stool numbers and weights were determined. As shown in Fig 1, stool numbers and weights were significantly decreased after treatment of lop, while 300 mg CO and FV-alone treated groups were shown to increase these fecal parameters compared with those in lop-treated group. Furthermore, combined administration of 300 mg CO and FV have the preventive effects against constipation more than the CO and FV-alone treated groups on constipated rats by lop. These results demonstrated that CO and FV, especially, combined treatment of CO and FV improved lop-induced constipation in rats through the enhancement of fecal parameters.

### Effects of CO and FV combined treatment on body weight and feeding behavior of constipated rats

**Fig 2:**
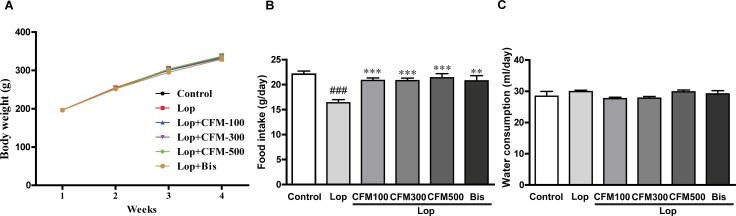
Body weight (BW) changes and feeding behavior during experimental procedure. BW (A) was measured once a week, and food intake (B) and water consumption (C) were daily measured in each group. N = 10 in each group. Data are expressed as mean ± SEM. Significance was measured by performing a one-way ANOVA followed by Bonferroni’s post-hoc test. ^###^ P < 0.001 vs. control. ** P < 0.01 and *** P < 0.001 vs. Lop-treated group. Lop, loperamide-induced constipated group; CFM 100, 300, and 500, CO and FV mixture 100, 300, and 500 mg/kg/day-treated group, respectively; Bis, bisacodyl-treated group.

To investigate whether CO and FV mixture treatment could affect the body weight and feeding behavior of constipated rats, body weight, food intake, and water consumption were measured during the experimental period. As shown in Fig 1, Body weight was steadily increased in each group. However, no differences in body weight changes in all groups were observed after administrations of lop, Co and FV mixture, and bisacodyl (bis), as a positive control drug (Fig 2A). Food intake was significantly decreased in lop-induced constipated rats (26.8% decrease vs. control group), while all doses of CO and FV mixture treatment were shown to similar levels with that of control group (Fig 2B). Finally, water consumption did not induce significant changes in all groups (Fig 2C). Collectively, these results demonstrated that CO and FV mixture treatment did not induce any alteration of body weight, food intake, and water consumption.

### Effects of CO and FV combined treatment on stool parameters of constipated rats

**Fig 3:**
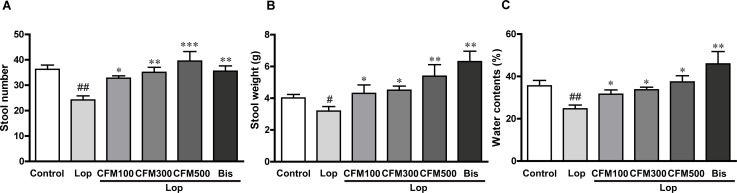
Effects of CO and FV mixture treatment on stool parameters of constipated rats. Stool number (A), stool weight (B), and water contents (C) were daily measured in each group. N = 10 in each group. Data are expressed as mean ± SEM. Significance was measured by performing a one-way ANOVA followed by Bonferroni’s post-hoc test. ^#^ P < 0.05 and ^##^ P < 0.01 vs. control. * P < 0.05, ** P < 0.01, and *** P < 0.001 vs. Lop-treated group. Lop, loperamide-induced constipated group; CFM 100, 300, and 500, CO and FV mixture 100, 300, and 500 mg/kg/day-treated group, respectively; Bis, bisacodyl-treated group.

To determine the laxative effects of CO and FV combined treatment on constipated rats, we examined the stool numbers, weights, and water contents in lop-, CO and FV mixture-, and bis-treated groups, respectively. Stool number was significantly reduced by 33.2% after treatment of lop compared with those in control group. Otherwise, CO and FV mixture treatments increased the stool numbers compared with those in lop-treated group (34.9%, 44.5%, and 63.2% increases in 100, 300, and 500 mg of CO and FV mixture -treated groups vs. lop-treated group, respectively; Fig 3A). Stool weight was also decreased in lop-treated group compared with that in control group (20.6% decrease vs. control group). However, this level was restored in all doses of CO and FV mixture-treated groups compared with that in lop-treated group (34.8%, 41.1%, and 69.0% increases in CO and FV mixture-100, 300, and 500 mg-treated groups vs. lop-treated group, respectively; Fig 3B). Furthermore, water contents of stool were shown to similar levels in control group when CO and FV mixtures were treated in constipated rats, while lop treatment reduced the water contents of stool (34.2% decrease vs. control group; Fig 3C). Similar results were found for bis treatment, as a positive drug.

### Effects of CO and FV combined treatment on intestine transit length and gastrointestinal transit ratio of constipated rats

**Fig 4:**
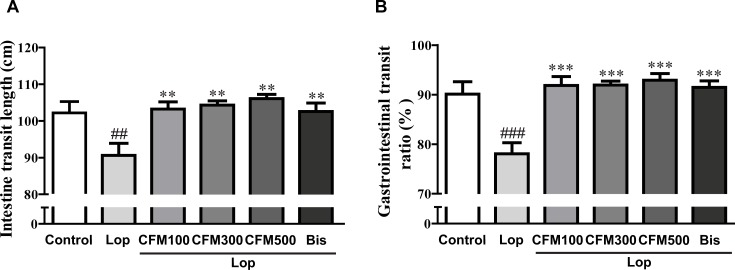
Effects of CO and FV mixture treatment on intestine transit length and gastrointestinal transit ratio of constipated rats. Intestine transit length (A) and gastrointestinal transit ratio (B) were observed in 30 min later after receiving barium sulfate. N = 10 in each group. Data are expressed as mean ± SEM. Significance was measured by performing a one-way ANOVA followed by Bonferroni’s post-hoc test. ^##^ P < 0.01 and ^###^ P < 0.001 vs. control. ** P < 0.01 and *** P < 0.001 vs. Lop-treated group. Lop, loperamide-induced constipated group; CFM 100, 300, and 500, CO and FV mixture 100, 300, and 500 mg/kg/day-treated group, respectively; Bis, bisacodyl-treated group.

As shown in Fig 4, the significant decreases in both intestine transit length and intestine transit ratio of barium sulfate were observed in lop-induced constipated rats compared with those in control group (11.3% and 13.4% decreases of intestine transit length and ratio in lop-treated group vs. control, respectively). Otherwise, these decreases were attenuated by CO and FV mixture treatment in lop-induced constipated rats, which were similar levels with those in control group.

### Effects of CO and FV combined treatment on histological properties of transverse colon in constipated rats

**Fig 5:**
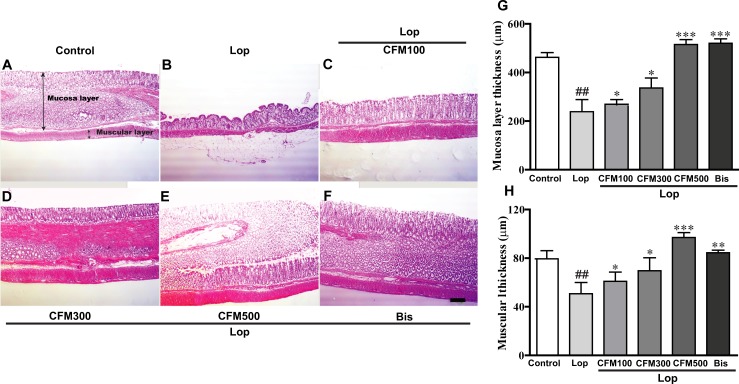
Effects of CO and FV mixture treatment on histological properties of transverse colon in constipated rats. H&E staining of transverse colon in lop-, CO and FV mixture-, and bis-treated groups (A-F). Scale bar, 100 μm. Mucosa layer (G) and muscular layer (H) thicknesses are presented as graphs. N = 5 in each group. Data are expressed as mean ± SEM. Significance was measured by performing a one-way ANOVA followed by Bonferroni’s post-hoc test. ^##^ *P* < 0.01 vs. control. ^*^ *P* < 0.05, ^**^ *P* < 0.01, and ^***^ *P* < 0.001 vs. Lop-treated group. Lop, loperamide-induced constipated group; CFM 100, 300, and 500, CO and FV mixture 100, 300, and 500 mg/kg/day-treated group, respectively; Bis, bisacodyl-treated group.

To determine whether CO and FV mixture treatment could alleviate the histopathological alterations of the transverse colon in constipated rats, we determined the morphology of the transverse colon by H&E staining. The results showed that the thicknesses of mucosa and muscular layers of transverse colon in lop-induced constipated rats were decreased compared with those in control group (48.4% and 36.2% decreases of mucosa and muscular layer vs. control group, respectively). However, CO and FV mixture treatment showed that the thicknesses of mucosa and muscular layers were maintained, with the most effective in 500mg/kg CO and FV mixture-treated groups compared with those in lop-treated group (15.8%, 40.9%, and 116% increases of mucosa layer and 20.0%, 37.3%, and 91% increases of muscular layer in CO and FV mixture-100, 300, and 500 mg-treated groups vs. lop-treated group, respectively; Fig 5). Additionally, similar results were found for bis treatment. Therefore, these results indicate that Co and FV mixture treatment could maintain the histological structures of transverse colon in constipated rats.

### Effects of CO and FV combined treatment on mAchRs and their downstream signaling pathway in constipated rats

**Fig 6:**
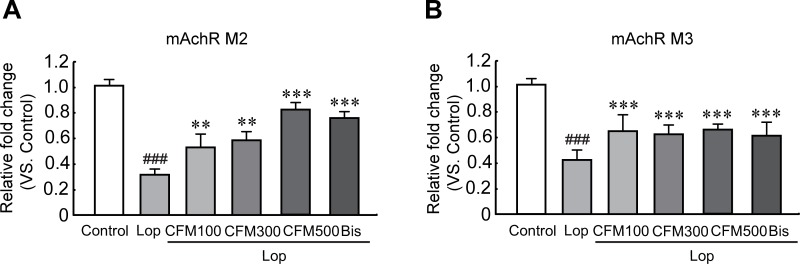
Effects of CO and FV mixture treatment on mRNA expression of mAchR M2 and M3 in the transverse colon. qRT-PCR analysis of mAchR M2 (A) and M3 (B) mRNA expression. N = 10 in each group. Data are expressed as mean ± SEM. Significance was measured by performing a one-way ANOVA followed by Bonferroni’s post-hoc test. ^###^ P < 0.001 vs. control. ** P < 0.01, and *** P < 0.001 vs. lop-treated group. Lop, loperamide-induced constipated group; CFM 100, 300, and 500, CO and FV mixture 100, 300, and 500 mg/kg/day-treated group, respectively; Bis, bisacodyl-treated group.

**Fig 7:**
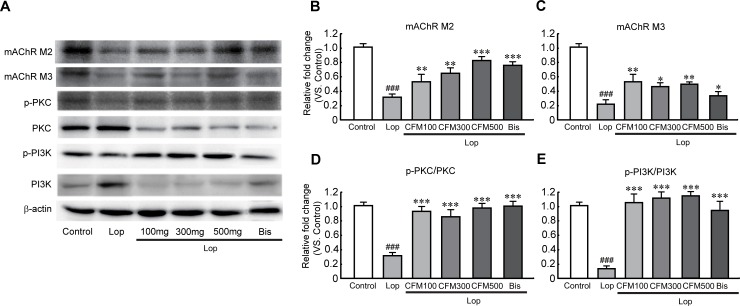
Effects of CO and FV mixture treatment on protein expression of mAchR M2 and M3 and PKC and PI3K as their downstream signaling pathway-related proteins in the transverse colon. N = 10 in each group. Western blot analysis of mAchR M2 and M3 (A-C) and phosphorylated and total of PKC and PI3K (D-F) protein expression. Data are expressed as mean ± SEM. Significance was measured by performing a one-way ANOVA followed by Bonferroni’s post-hoc test. ^###^ P < 0.001 vs. control. * P < 0.05, ** P < 0.01, and *** P < 0.001 vs. Lop-treated group. Lop, loperamide-induced constipated group; CFM 100, 300, and 500, CO and FV mixture 100, 300, and 500 mg/kg/day-treated group, respectively; Bis, bisacodyl-treated group.

To determine whether CO and FV mixture treatment could affect the regulations of gene expression related to the muscle contraction, we performed the quantitative RT-PCR (qRT-PCR) of two mAchRs (mAchR M2 and M3), which play a role for smooth muscle contraction, of the transverse colons in constipated rats. The mRNA expression levels of mAchR M2 and M3 were significantly reduced in lop-induced constipated rats compared with those in control group, but the reduced levels were substantially inhibited when treated CO and FV mixture and bis (Fig 6). In addition, these protein expression patterns were also decreased in lop-treated group, while the reduced mAchR M2 protein levels were gradually increased as concentration of CO and FV mixture was increased and mAchR M3 protein levels were significantly increased in all CO and FV mixture treated group compared with those in lop-treated group (Fig 7A, 7B and 7C). Moreover, phosphorylation of PKC and PI3K, as the mAchRs downstream signaling pathway, were significantly reduced by lop treatment. However, these reductions were dramatically prevented by CO and FV mixture treatment as similar values in control group (Fig 7A, 7D and 7E). Hence, these results indicate that the laxative effects of CO and FV mixture may act through the regulation of mAchR M2 and M3 and their downstream signaling pathway.

## Discussion

Recently, herbal plants and natural products have been attracted much attention to develop the therapeutic drugs for the treatment of constipation due to their effectiveness and safety properties [28]. Accordingly, their laxative effects and underlying mechanisms on constipation have been intensively studied [29]. CO and FV have the beneficial effects against many diseases, including hepatoprotection [30, 31], neuroprotection [32], and antioxidant [33] [34]. CO exhibited the preventive effect against hyperlipidemia by decreasing serum low-density lipoprotein and triglycerides [35]. Additionally, FV has the therapeutic effects against cardiovascular disorders by reducing blood pressure without affecting the heart functions [36]. The previous studies have also demonstrated that FV has the anti-cancer effects in breast (MCF-7) and liver (Hepg) cancer cell lines [37]. In the present study, we sought to determine the laxative effects of CO and FV mixture in lop-induced constipated rats.

In this study, we first demonstrated that cotreatment of CO and FV have preventive effects against the constipation on lop-treated rats. Indeed, lop-induced constipation is well established and widely used as a model of spastic constipation [38]. Lop suppresses the water secretion and peristalsis in the colon, which causes to delay stool evacuation time and intestine luminal transit [39]. Consequently, these states directly affect the reduction of feed and water intakes in lop-exposed rats. Therefore, food intake and water consumption are considerable factors for the evaluation of constipation [28]. In our study, the analysis of the food intake revealed that the treatment of lop significantly decreased the food intake in rats, although the water consumption did not change among groups. Otherwise, administration of CO and FV mixture increased the food intake in lop-induced constipated rats, which were similar levels in control group.

In constipation, the obstacle of water absorption causes to decrease fecal discharges and delay fecal pellet transit in the large intestine. These alterations of fecal properties have been used as indicators of constipation symptoms and as indices of therapeutic effects against constipation [40]. Therefore, improvements of discharged fecal parameters, including increases of stool numbers and soften stools, and intestinal transit time, is one of the important strategy for the constipation treatment [41, 42]. Previous studies showed dramatically decreased the stool-related parameters, including stool numbers, weights and water contents in lop-treated rats [12, 43]. Consistently, our data demonstrated that these parameters were significantly reduced in lop-treated group. Importantly, treatment of CO and FV mixture enhanced the fecal properties in constipated rats, as similar with the levels of normal rats. In addition, CO and FV mixture treatment in lop-induced constipated rats effectively recovered gastrointestinal transit ratio of barium sulfate. Collectively, these findings indicate that treatment of CO and FV mixture may have laxative potential against constipation through the improvement of colonic motor activity, feces excretion, and release of fluids in the intestine.

Histological studies have demonstrated that lop treatment caused the significant alterations in the transverse colon such as decreases of both mucosa and muscular layers. Previous studies have reported that constipation was accompanied by the markedly decreases of the colonic mucosa and muscular layers [12, 44]. Therefore, the preventive effects against these pathological states of colon were direct evidence of laxative effects of therapeutic drug candidates. As expected, treatment of CO and FV mixture dramatically increased mucosa and muscular layers of colon in constipated rats.

mAchRs belongs to the Ach receptors that are expressed in many cells (such as, neurons, heart, and smooth muscle cells) in the body and their roles are involved in many cell functions, including medication of cholinergic transmission, immune responses, and regulation of cell growth [45]. mAchR M1, M2, and M3 are expressed in the colon and have crucial roles for the intestinal activities, particularly motility and secretion [46]. Previous studies were reported to the decreases of mAchRs and their downstream proteins, such as PKC and PI3K in constipation [13]. Regarding with this, we demonstrated that both mRNA and protein expression of mChR M2 and M3 were significantly decreased after lop treatment. Particularly, these mAchR M2 and M3 decreases were dramatically recovered by Co and FV mixture treatment. Furthermore, protein expression of PKC and PI3K as downstream signaling pathway was revealed that CO and FV mixture treatment dramatically prevent the decreases levels after lop administration. Taken together, we further confirmed that laxative effect of Co and FV cotreatment may mediate by activation of AchR M2 and M3-related signaling pathway in constipated rats.

In conclusion, our study suggests that CO and FV mixture treatment has the laxative effect by recovering stool-parameters, colonic morphology, and activation of mAchRs and their downstream signaling pathway in constipation. Furthermore, our study provides that CO and FV cotreatment could be considered as a therapeutic drug candidate for the prevention or treatment of constipation.
