# Correction: The combination of *Cassia obtusifolia* L. and *Foeniculum vulgare* M. exhibits a laxative effect on loperamide-induced constipation of rats

**DOI:** 10.1371/journal.pone.0202259

**Published:** 2018-08-09

**Authors:** Seung Hee Jang, Dong Kwon Yang

In the Materials and Methods section, there is an error in the third sentence under the heading: “Preparation of CO and FV.” The correct sentence is: The dried CO powders were then twice extracted in 70% ethanol (powder sample/70% ethanol, 1:8) at 70 °C for 3 h. For the FV extraction, the dried FV samples were extracted three times with distilled water (powder sample/distilled water, 1:10) at 95 °C for 1.5 h.
